# The American and Colonial Journals: Midwifery

**Published:** 1839-10

**Authors:** 


### MIDWIFERY.

### *On the Temperature of the Vagina and Os Uteri during Labour.* By Prof. DUNGLISON, of Philadelphia.

THE remark of Dr. Granville, that the temperature of the uterine system during parturition, sometimes rises as high as 120° of Fahrenheit's scale, has always struck us as needing further confirmation. We have often been impressed with the seemingly elevated temperature of the vagina under these circumstances, but have always suspected inaccuracy in the observations of Dr. Granville, not only because the temperature he indicates is so much higher than has ever been noticed in any condition of the system, or of any organ, but because the results of our own experiments have not shown that the temperature is *really* much elevated in the cases in question.

The following results of observations made at our request by Dr. Barnes, one of the Senior Resident Physicians of the Philadelphia Hospital, Blockley, so far as they go, confirm our own. They likewise exhibit the ratio of the pulsations of the maternal and the fœtal heart at the times of observation.

OBS. I.—Pulse, 84; fœtal heart, 130; temp. within labia, 100°; temp. at os uteri, 100°.

This is the *average* result of a series of thermometric observations, made during a space of twenty-five minutes; six hours after the commencement of true labour pains, and one hour previous to the delivery of the child. The patient was in labour with her first child, of an exanguious habit—with a hereditary predisposition to phthisis.

OBS. II.—Pulse, 72; fœtal heart, 120; temp. at labia, 100; temp. within os uteri, 102.

The result of a single observation made, in the case of T—— A——, twelve hours after the commencement of regular and severe, but not propulsive pains. The patient is stout and muscular, of short stature, and of intemperate habits. A few minutes after making the first observation, the pains ceased entirely, and did not recur until twenty-four hours after.

OBS. III.—Pulse, 73; fœtal heart, 128; temp. at labia, 105; temp. within os uteri, 106.

The *average* result of a series of observations made during a space of two hours, commenced fourteen hours after first labour pains, and terminating with the delivery of the placenta. The patient was of a full and corpulent habit, and in labour with her first child. *American Med. Intelligencer. Feb.* 1839.

